# Anti-Proliferative and Anti-Migratory Activities of Hispidulin on Human Melanoma A2058 Cells

**DOI:** 10.3390/biom11071039

**Published:** 2021-07-16

**Authors:** Chi-Jen Chang, Yen-Ling Hung, Ting-Chen Chen, Hsin-Ju Li, Yuan-Hsin Lo, Nan-Lin Wu, Der-Chen Chang, Chi-Feng Hung

**Affiliations:** 1 School of Medicine, Fu Jen Catholic University, New Taipei City 24205, Taiwan; 054317@gmail.com; 2 Division of Pediatric Surgery, Shin Kong Wu Ho-Su Memorial Hospital, Taipei 111045, Taiwan; 3 Graduate Institute of Biomedical and Pharmaceutical Science, Fu Jen Catholic University, New Taipei City 24205, Taiwan; irishung0919@gmail.com (Y.-L.H.); 409466019@mail.fju.edu.tw (T.-C.C.); 054317@mail.fju.edu.tw (H.-J.L.); 4 Department of Dermatology, Fu Jen Catholic University Hospital, Fu Jen Catholic University, New Taipei City 24205, Taiwan; A00781@fjuh.fju.edu.tw; 5 Department of Medicine, Mackay Medical College, New Taipei City 25245, Taiwan; d94443002@ntu.edu.tw; 6 Department of Mathematics and Statistics and Department of Computer Science, Georgetown University, Washington, DC 20057, USA; chang@georgetown.edu; 7 Department of Fragrance and Cosmetic Science, Kaohsiung Medical University, Kaohsiung 80708, Taiwan; 8 PhD Program in Pharmaceutical Biotechnology, Fu Jen Catholic University, New Taipei City 24205, Taiwan

**Keywords:** melanoma, hispidulin, natural product

## Abstract

Melanoma represents less than 5% of skin cancers, but is the most lethal, mainly because of its high-metastatic potential and resistance to various therapies. Therefore, it is important to develop effective treatments, especially chemotherapeutic drugs with cytotoxicity, anti-metastaticity, and few side effects. One such natural product is hispidulin, a flavone distributed in plants of the *Asteraceae*. Previous studies have demonstrated that hispidulin has various pharmacological benefits, such as anti-tumor, anti-inflammation, and anti-allergic effects. This study aims to explore the effects of hispidulin against melanoma in vitro and in vivo. Results revealed that hispidulin selectively decreased the cell viability of A2058 cells in a dose- and time-dependent manner. Hispidulin induced cells accumulated in the sub-G1 phase via activating caspase 8 and 9, increased cleaved caspase 3, and cleaved PARP expression. Hispidulin was able to decrease AKT and ERK phosphorylation, which facilitated cell growth and survival. Moreover, hispidulin promoted reactive oxygen species generation in cells and suppressed cell migration through downregulated matrix metalloproteinase-2 expression. Hispidulin significantly inhibited tumor growth in a xenograft model. Based on these results, hispidulin produces its anti-melanoma effects by inducing cancer cell apoptosis and reducing its migration. Therefore, we suggest hispidulin as a potent therapeutic candidate for melanoma treatment.

## 1. Introduction

The number of patients suffering from skin cancer worldwide has recently been increasing every year [1]. Generally, skin cancer is more common in the elderly population, but the age group of patients with skin cancer has tended to decline in recent years, with cases occurring in patients ranging from age 35 to 65 [2]. Skin cancer can be divided into two categories based on the source of cancer cells, namely non-melanoma cancer and melanoma cancer, of which the incidence rate of the former is higher than that of the latter. Melanoma cancer originates from melanocytes. It has a low prevalence, but a high lethality rate. About 80% of skin cancer deaths are attributable to melanoma cancer [3]. Melanoma cancer is a highly invasive and metastatic malignant tumor. Most patients discover cancer only when they have obvious metastatic symptoms in the middle and late stages. Malignant melanoma easily metastasizes or spreads through the blood or lymphatic system. Once the metastasis spreads to other tissues or organs, it is called metastatic malignant melanoma, which cannot be cured by surgical resection. Therefore, more than 90% of patients die because cancer cells have metastasized to other tissues and organs [4]. General chemotherapy drugs, radiotherapy, and immunotherapy have a limited effect on it. It is very difficult to treat, and its mortality rate is quite high. So far, there is no effective drug or strategy to control or treat metastatic melanoma. Determining how to extend the effective duration of chemotherapy drugs and reduce their side effects to improve the quality of life of patients or prolong the survival of patients is important, yet extremely difficult. Improving the effectiveness of chemotherapy through the research and development of traditional herbs or natural medicines is important.

Traditional Chinese herbal medicine (TCM) has been used for thousands of years, but research work on scientifically validating active ingredients and pharmacology has flourished only in the past one or two decades. Chinese herbal medicine has been used throughout human history, and currently more than 40% of prescription medicines in the world are derived mainly from herbal medicines [5]. There are also accumulated research results for the use of herbal medicine against malignant skin melanoma. For example, curcumol is a polyphenol compound isolated from the ethanol extracts of *Curcuma wenyujin*. It attenuated melanoma progression, concomitantly suppressed ERK/NF-ĸB signaling, and promoted miR-152-3p expression to inactivate the c-MET/PI3K/AKT signaling pathway [6]. Herbacetin is a flavonol compound that is found in plants such as flaxseed and ramose scouring rush herb. It effectively attenuated TPA-induced skin cancer development and also exhibited therapeutic effects against solar-UV-induced skin cancer and melanoma growth in vivo [7]. Shikonin derivatives in the *Lithospermum erythrorhizon* are responsible for anticancer activity [8]. Ethosomes containing a combination of berberine chloride and evodiamine are a promising delivery system for potential use in melanoma therapy [9]. Hispidulin (4′,5,7-trihydroxy-6-methoxyflavone) is a flavone derivative isolated mainly from *S. involucrata*, a medicinal plant traditionally used in oriental medicine [10]. In recent years, hispidulin exhibited various pharmacological effects, including anti-inflammatory, neuroprotective and anti-tumor activities [11,12,13,14,15,16,17,18,19,20,21,22]. The present study aims to investigate the anticancer effects of hispidulin in melanoma cells. Our current data reveal that the inhibitory effect of hispidulin on the proliferation and migration of A2058 cells may be related to the inhibition of AKT and ERK pathways. We believe that hispidulin has the potential to be a natural compound for the treatment of melanoma.

## 2. Materials and Methods

### 2.1. Cell Culture

A2058 human melanoma cells, basal cell carcinoma cells (BCC), and keratinocytes were used for research. A2058 cells were purchased by the Food Industry Research and Development Institute (FIRDI) in Taiwan. BCC and Human immortalized keratinocytes (HaCaT cells) were a gift from J.Y. Fang, Chang Gung University, Taoyuan, Taiwan. Human primary epidermal keratinocytes (NHEK) are derived from human foreskin. After obtaining permission from the Institutional Review Board (#13MMHIS022), Mackay Memorial Hospital, New Taipei City, Taiwan provided the foreskin. The cells (A2058, BCC, and HaCaT) were cultured in Dulbecco’s Modified Eagle Medium (DMEM, GibcoBRL, Invitrogen, Carlsbad, CA, USA) and supplemented with a 10% fetal bovine serum (FBS; GibcoBRL, Invitrogen, Carlsbad, CA, USA) and an antibiotic-antimycotic solution (GibcoBRL, Invitrogen, Carlsbad, CA, USA). The NHEK were cultured in Keratinocyte-SFM (Gibco BRL, Invitrogen, Carlsbad, CA, USA) and supplemented with recombinant epidermal growth factor (0.1–0.2 ng/mL), bovine pituitary extract (20–30 mg/mL), and 1% penicillin/streptomycin. The cells were cultured in a cell incubator at 37 °C and 5% CO_2_.

### 2.2. Cytotoxic Assays

Cell survival was determined by MTT and crystal violet colorimetric assays. An MTT assay is a versatile and popular assay to assess mitochondrial activity, whereas crystal violet is a basic dye that can bind to the nucleic acid in the cell nucleus to dye the nucleus blue. Cells were evenly planted in a 24-well culture plate at a concentration of 2 × 10^4^/well, then 500 µL of full medium was added to each well, and the wells were placed in a 37 °C, 5% CO_2_ incubator for one day. The cells were briefly treated with various concentrations of hispidulin. For the MTT assay, the 3-(4,5-Dimethylthiazol-2-yl)-2,5-diphenyltetrazolium bromide (MTT) was then added, followed by incubation at 37 °C for 4 h. The supernatant was removed, and the blue-purple crystals were dissolved with DMSO. The absorbance was measured at a wavelength of 550 nm by an ELISA reader (Molecular Devices, San Jose, CA, USA). For the crystal violet assay, methanol was added to fix for 30 min and then removed after fixation and air dried. Next, 300 µL of 0.1% crystal violet aqueous solution was added for one hour. Finally, the crystal violet was dissolved with 500 µL/well of 33% acetic acid solution. The absorbance was measured at a wavelength of 550 nm by an ELISA reader.

### 2.3. Flow Cytometry Analysis

Propidium iodide (PI) staining was used to analyze the cell cycle. A2058 cells were evenly seeded in a 25 T flask. Blank solvent and 10, 30, and 50 µM of hispidulin were added to the concentrations, which were then placed in a 37 °C, 5% CO_2_ incubator for 24 h. Next, the cells were harvested and washed with PBS, then fixed overnight in methanol at 4 °C and stained with PI solution (including 100 µg/mL PI, 0.1% TritonX-100, and 200 µg/mL RNase). The DNA content of the cells was determined by flow cytometry (Partec; CyFlow^®^ ML, Sysmex Partec, Germany).

Apoptosis was analyzed using Annexin V/PI double staining. The cells were seeded in a 25 T flask. The various concentrations of hispidulin (10, 30, and 50 µM) were added to the 25 T flask, which was then placed in a 37 °C, 5% CO_2_ incubator for 24 h. The supernatant and cells were collected and placed in a centrifuge at 1100 rpm, 25 °C, for 5 min. The cells (4 × 10^5^) were counted and suspended in a 100 µL Annexin V/PI solution (Strong Biotech Co., Taiwan). The solution contained an Annexin V binding buffer, Annexin V-FITC, and PI. The reaction was carried out at room temperature in the dark for 15 min. Finally, an Annexin V binding buffer was added and analyzed by a flow cytometer (Partec; CyFlow^®^ ML, Sysmex Partec, Germany).

### 2.4. Intracellular ROS Detection

2′,7′-Dichlorodihydrofluorescein diacetate (DCFH-DA) was used to detect the content of ROS in cells. The cells were seeded in a 6-well plate and placed in a 37 °C, 5% CO_2_ incubator. Blank solvent, hispidulin, and N-acetylcysteine were added to 6 wells and placed in a 37 °C, 5% CO_2_ incubator for 24 h. Then, 5 mM DCFH-DA solution was added, and the wells were placed in the dark for 30 min at 37 °C. The cells were collected, suspended the in PBS, and examined by flow cytometer.

### 2.5. Cell Migration

The wound healing assay was used to analyze the migration of the cells. The cells were evenly seeded in a 6-well culture plate and placed in a 37 °C, 5% CO_2_ incubator for one day. Entire confluent A2058 sheets were scratched with 200 µL tips. Then, the status of cell migration was observed for 24 and 48 h. The quantitative assessment of the number of cells in the denuded zone is as follows. The cell wound closure rate was calculated using the following equation: Wound closure = [1 − (wound area at Tt/wound area at T0)] × 100, where Tt is the time passed since wounding and T0 is the time the wound was created. In addition, transwell was also used to study the effects of A2058 cell migration. The cells (1.5 × 10^4^/200 µL) were added to the transwell that had been coated on the collage and incubated for 5 h. Cells that did not migrate through the hole were removed with a cotton swab. Cells under the transwell were fixed with paraformaldehyde and stained with migration dye for one hour. By counting the cells passing through transwell, we examined the effect of different concentrations of hispidulin on the migration ability of A2058 cells.

### 2.6. Western Blotting Analysis

The process was performed as described previously [23]. Samples were washed with PBS and lysed in a radioimmunoprecipitation assay buffer. After sonication, the lysate was centrifuged (13,200 rpm for 10 min at 4 °C), and supernatant was transferred to a tube. Equal amounts of soluble protein were loaded and electrophoresed on SDS-10% polyacrylamide gels, electroblotted onto PVDF (polyvinylidene difluoride) membranes, and then probed using primary antibodies against Cyclin D1, Cleaved PARP, Caspase 3,8,9, Beclin-1, LC3B, p62, AKT, ERK, JAK2, MMP-2, or GAPDH (Cell Signaling Technology). Immunoblots were detected by enhanced chemiluminescence (ECL, Chemiluminescence Reagent Plus from PerkinElmer, Waltham, MA, USA). The intensity of the bands obtained was quantified using the ImageJ software. ImageJ bundled with 64-bit Java 1.8.0_172 is a Java-based (runs on all operating systems) freeware by Wayne Rasband from National Institute of Health (USA) and is available for download at: http://rsb.info.nih.gov/ij/ (accessed on 16 July 2021).

### 2.7. Xenograft Model

All animal experiments in this study were approved by the Institutional Animal Care and Use Committee of Fu Jen Catholic University (the permission number: A10703). A2058 cells were prepared with a concentration of 1 × 10^6^/0.1 mL in PBS (PBS:matrigel = 1:1). The cells were inoculated on the right back and randomly divided into two groups, the control group and the drug group. On the 7th day after cell inoculation (tumor size reached 100–200 mm^3^), mice were administered intraperitoneal injections with DMSO in PEG 400 (vehicle control) or 40 mg/kg hispidulin, once a day for 19 consecutive days. Clinical symptoms (fur, eyes, feces) were observed daily. Food, water, mouse body weight, and tumor volume were monitored every 3 days. The tumor volume was calculated using the following formula: (length of tumor) × (width of tumor)^2^ × 0.5. At the end of the final experiment, the mice’s weight was measured and the appearance of tissues—including lung, liver, and heart—were observed. Finally, the tumor was removed and processed through experiments, such as weighing the tumor and hematoxylin and eosin (H&E) staining with paraffin sections.

### 2.8. Statistical Analysis

Statistical analysis was performed using PRISM 6.0 software. The experimental data were expressed as mean ± standard error (SE) and analyzed by an unpaired, two-tailed Student’s t-test. Differences between groups, using the group without any treatment as the control group, were used to evaluate the treatment of hispidulin. A *p*-value less than 0.05 is considered statistically significant. The following notations are used: * *p* < 0.05 and ** *p* < 0.01.

## 3. Results

### 3.1. Cytotoxicity of Hispidulin to A2058 Cells and Keratinocyte

**Figure 1:**
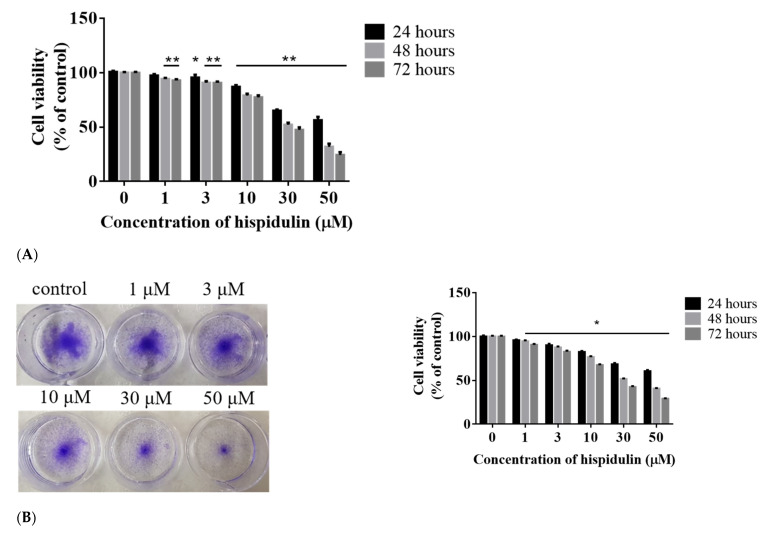

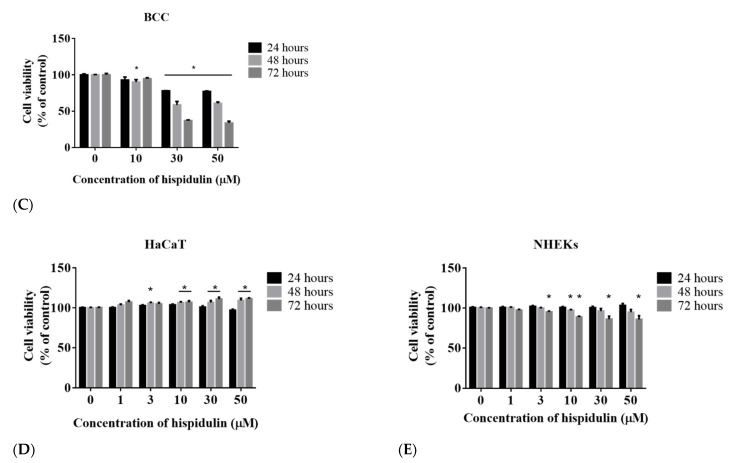
Cytotoxicity of hispidulin on A2058 cells, BCC cells, and keratinocytes. A2058 cells were treated with indicated concentrations (0, 1, 3, 10, 30, and 50 µM) of hispidulin for 24, 48, and 72 h, and cell survival was determined by the (**A**) MTT assay and (**B**) crystal violet assay. (**C**) BCC cells were treated with indicated concentrations (0, 10, 30, and 50 µM) of hispidulin for 24, 48, and 72 h, and cell survival was determined by the MTT assay. (**D**,**E**) Two kinds of keratinocytes (HaCaT and NHEKs) were treated with indicated concentrations (0, 1, 3, 10, 30, and 50 µM) of hispidulin for 24, 48, and 72 h, and cell survival were determined by the MTT assay. Data are presented as percentage of control and mean ± SE. * *p* < 0.05 compared with the untreated control group (n = 5).

In order to explore the potential use of hispidulin in melanoma treatment, A2058 cells were treated with low to high concentrations (1–50 µM) of hispidulin or vehicle for 24, 48, and 72 h, and MTT and crystal violet assays were used. The results showed that hispidulin had a dose- and time-dependent cytotoxicity to A2058 cells. The MTT assay showed that A2058 cells were treated with different hispidulin concentrations of 1, 3, 10, 30, and 50 µM for 24 h, and cell viability levels were 97.4 ± 1.4%, 95.6 ± 2.6%, 92.5 ± 0.8%, 78.7 ± 0.8%, and 73.7 ± 0.9%. After 48 h, the cell viability decreased to 94.4 ± 1.0%, 90.8 ± 1.5%, 78.9 ± 1.8%, 52.2 ± 1.9%, and 31.9 ± 1.0%. When the cells were exposed to hispidulin for 72 h, the cell survival rate decreased significantly to 93.0 ± 1.1%, 90.7 ± 1.2%, 77.4 ± 1.9%, 47.5 ± 2.3%, and 24.5 ± 2.6% (Figure 1A). The crystal violet assay was also used to detect the number of A2058 viable cells. The cytotoxicity of hispidulin is also dose- and time-dependent. After treatment with a high concentration of hispidulin (50 µM) for 24 h, the cell death rate was about 30%. When the action time was extended to 48 or 72 h, the cell death rate increased to 60% and 70% (Figure 1B). In order to further prove the potential of hispidulin in skin cancer, we also performed the cytotoxic effect of hispidulin in basal cell carcinoma (BCC). As shown in Figure 1C, hispidulin also has a cytotoxic effect in BCC. In order to further evaluate the toxicity of hispidulin, hispidulin was used in different concentrations (1, 3, 10, 30, and 50 µM) on human keratinocytes (HaCaT and human keratinocyte NHEKs). We noted that hispidulin did not affect the cell viability of keratinocytes (Figure 1D,E). Based on the above, hispidulin selectively inhibits the growth of A2058 melanoma cells and shows a low cytotoxic effect on normal cells.

### 3.2. Hispidulin Promoted Cell Cycle Arrest in A2058 Melanoma Cells

**Figure 2:**
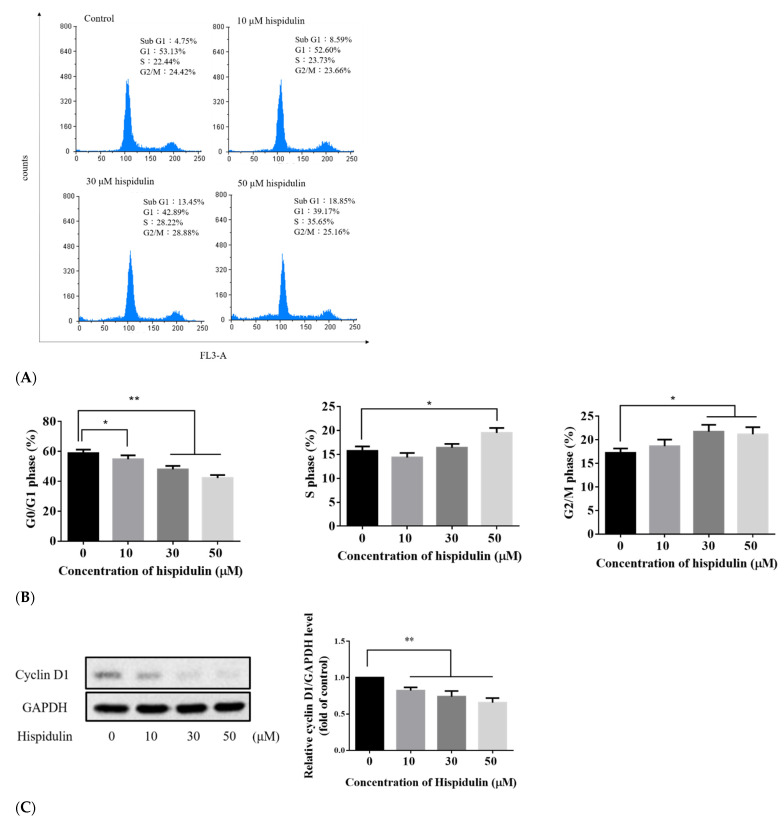
(**A**) Effect of hispidulin on the cell cycle in A2058 cells. A2058 cells were incubated with various concentrations (0, 10, 30, and 50 µM) of hispidulin for 24 h followed by staining with PI for flow cytometric analysis to determine cell cycle distribution. Results reveal the number of cells/channel (*y*-axis) versus DNA content (*x*-axis). The values shown present the percentage of cells in the indicated phases of the cell cycle and the cell distribution in different phases of the cell cycle. (**B**) Statistical analysis of hispidulin-treated A2058 cell cycle distribution. (**C**) Effect of hispidulin on cell cycle-related protein in A2058 cells. Expression levels of cyclin D1 in A2058 cells treated with hispidulin (0, 10, 30, and 50 µM) for 24 h were examined by Western blot assay. GAPDH was used as an internal control. Data were presented as fold of control and mean ± SE. * *p* < 0.05 and ** *p* < 0.01 compared with the untreated control group (n = 6).

Previous studies have indicated that hispidulin can cause the G0/G1 phase cell cycle arrest in human renal cell carcinoma, glioblastoma, liver cancer, gallbladder cancer, and acute myeloid leukemia [16,18,20,24,25]. In gastric cancer, it is arrested in G1/S phase [26]. In order to further explore the mechanism of hispidulin inhibiting cell growth, the cell cycle distribution of A2058 cells treated with hispidulin was analyzed. The cell cycle was analyzed by flow cytometry. Compared with the control group, the number of cells in the G0/G1 phase decreased with the increase of hispidulin concentration, and accumulated in the S or G2/M phase (Figure 2A,B). Cyclin D1 plays a regulatory role in the transition from G1 to G1/S. The effect of hispidulin on the performance of cyclin D1 was further evaluated. The results show that the effect of hispidulin will reduce the expression of cyclin D1 in A2058 cells in a dose-dependent manner (Figure 2C). These results indicated that cyclin D1 is involved in hispidulin-mediated cell cycle arrest events.

### 3.3. Hispidulin Induced A2058 Cell Apoptosis Not Autophagy

**Figure 3:**
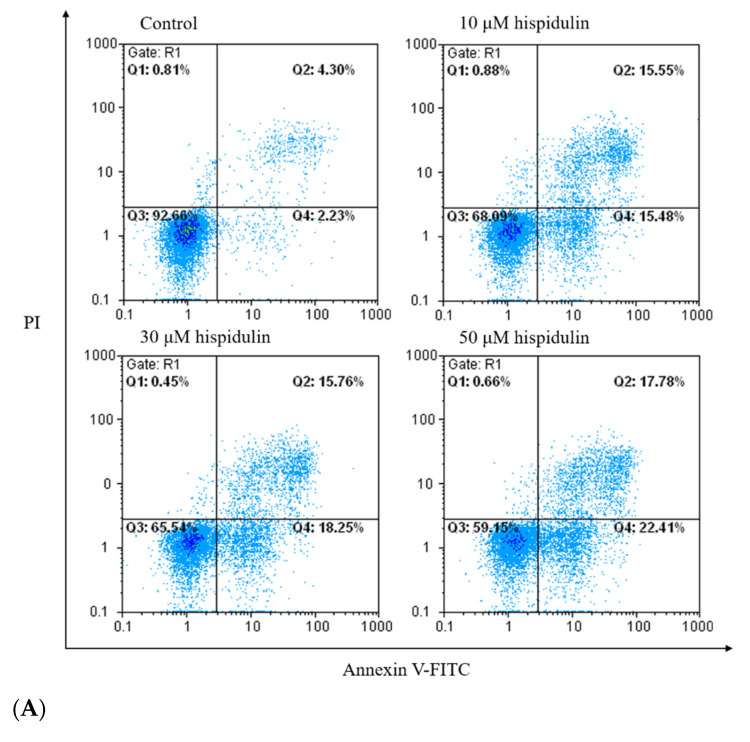

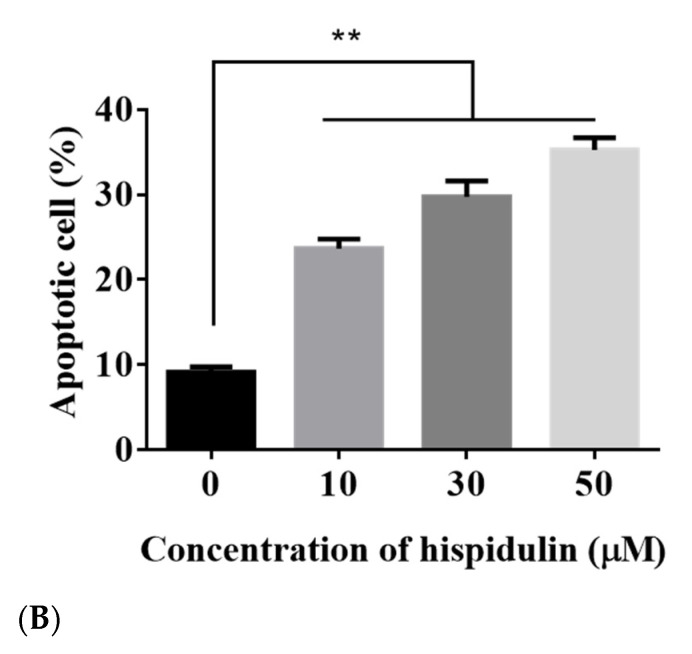
(**A**) The effect of hispidulin on cell apoptosis in A2058 cells as determined by flow cytometry. A2058 cells were treated with indicated concentrations (0, 10, 30, and 50 µM) of hispidulin for 24 h followed by double-staining with Annexin V/FITC and PI for flow cytometric analysis to determine the proportion of apoptotic cells. The flow cytometry profile presents Annexin V-FITC (*x*-axis) and PI staining (*y*-axis). The values represent the percentage of cells in each of the four quadrants (lower left quadrant: viable cells, upper left quadrant: necrotic cells, lower right quadrant: early-stage apoptotic cells, and upper right quadrant: late-stage apoptotic cells). (**B**) The apoptotic cells were quantified with early-stage and late-stage apoptotic cells. Data are presented as percentage of control and mean ± SE, with ** *p* < 0.01 compared with the control group (n = 6).

**Figure 4:**
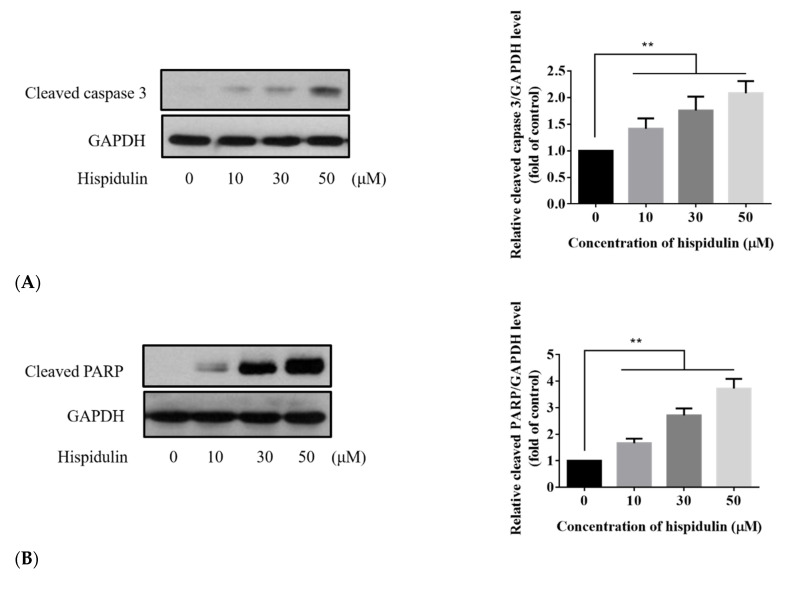

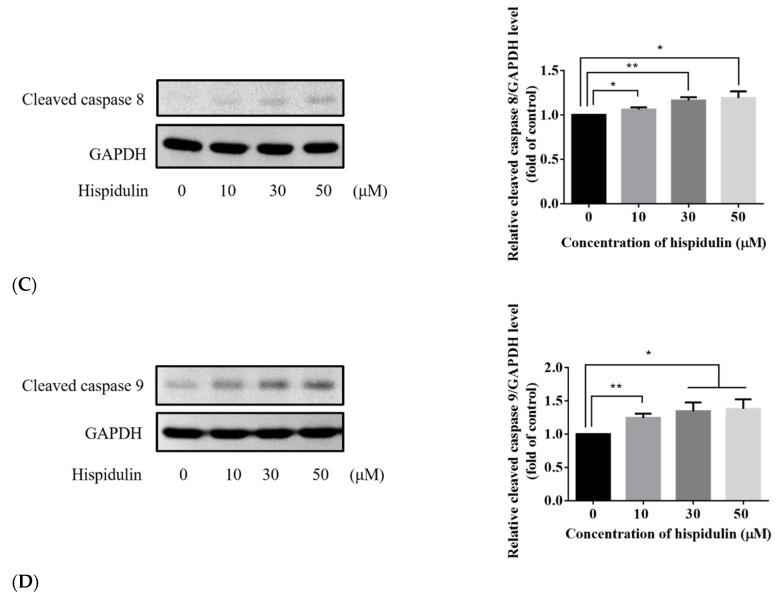
Expression levels of (**A**) cleaved caspase 3, (**B**) cleaved PARP, (**C**) cleaved caspase 8, and (**D**) cleaved caspase 9 in A2058 cells treated with indicated concentration of hispidulin or DMSO (control) for 24 h were examined by Western blot assay. GAPDH was used as an internal control. Data are presented as fold of control and mean ± SE, with * *p* < 0.05 and ** *p* < 0.01 compared with the control group (n = 5).

**Figure 5:**
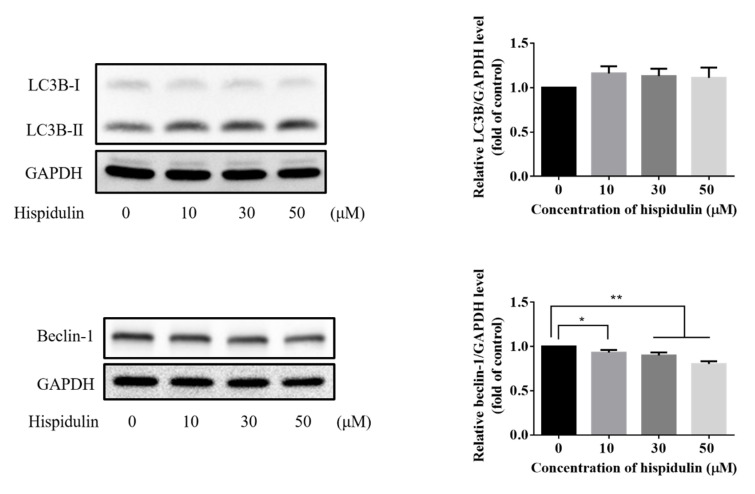

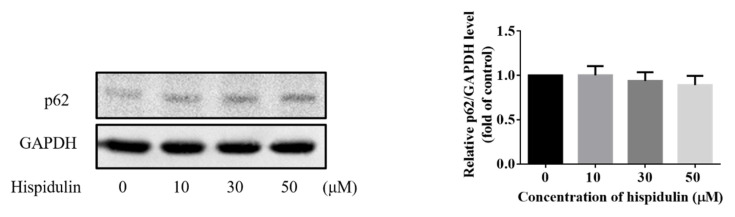
The expression of LC3B-I/LC3B-II, Beclin-1, and p62/SQSTM (p62) were assessed by Western blotting in A2058 cells treated with the indicated concentration of hispidulin or DMSO (control) for 24 h. Data were presented as fold of control and mean ± SE, with * *p* < 0.05 and ** *p* < 0.01 compared with the untreated control group (n = 5).

In order to further explore the potential mechanism of hispidulin inhibiting the growth of A2058 cells, we also analyzed the phenomenon of apoptosis. Phosphatidylserine turning to the outside of the cell membrane is characteristic of early apoptosis. The results showed that hispidulin induced apoptosis with treatments of hispidulin (10, 30, and 50 µM) for 24 h in A2058 cells. With the increase of hispidulin concentration, the number of apoptotic cells (including early apoptotic cells and late apoptosis) increased (Figure 3A,B). Cleaved caspase 3 and its substrate cleaved PARP are markers of apoptosis. We found that the expression of both cleaved caspase 3 and cleaved PARP increased significantly in a dose-dependent manner with treatments of hispidulin (Figure 4A,B). Furthermore, we found that hispidulin could significantly increase the performance of cleaved caspase 8 and cleaved caspase 9 in a dose-dependent manner (Figure 4C,D). Moreover, we evaluated related proteins involved in autophagy, including LC3B-I/LC3B-II, Beclin-1, and p62/SQSTM 1 (p62), but found that the expression levels were not significantly affected by hispidulin treatment. However, Beclin-1 slightly decreased in scientific significance (Figure 5).

### 3.4. Hispidulin Inhibited AKT and ERK Signaling Pathways

**Figure 6:**
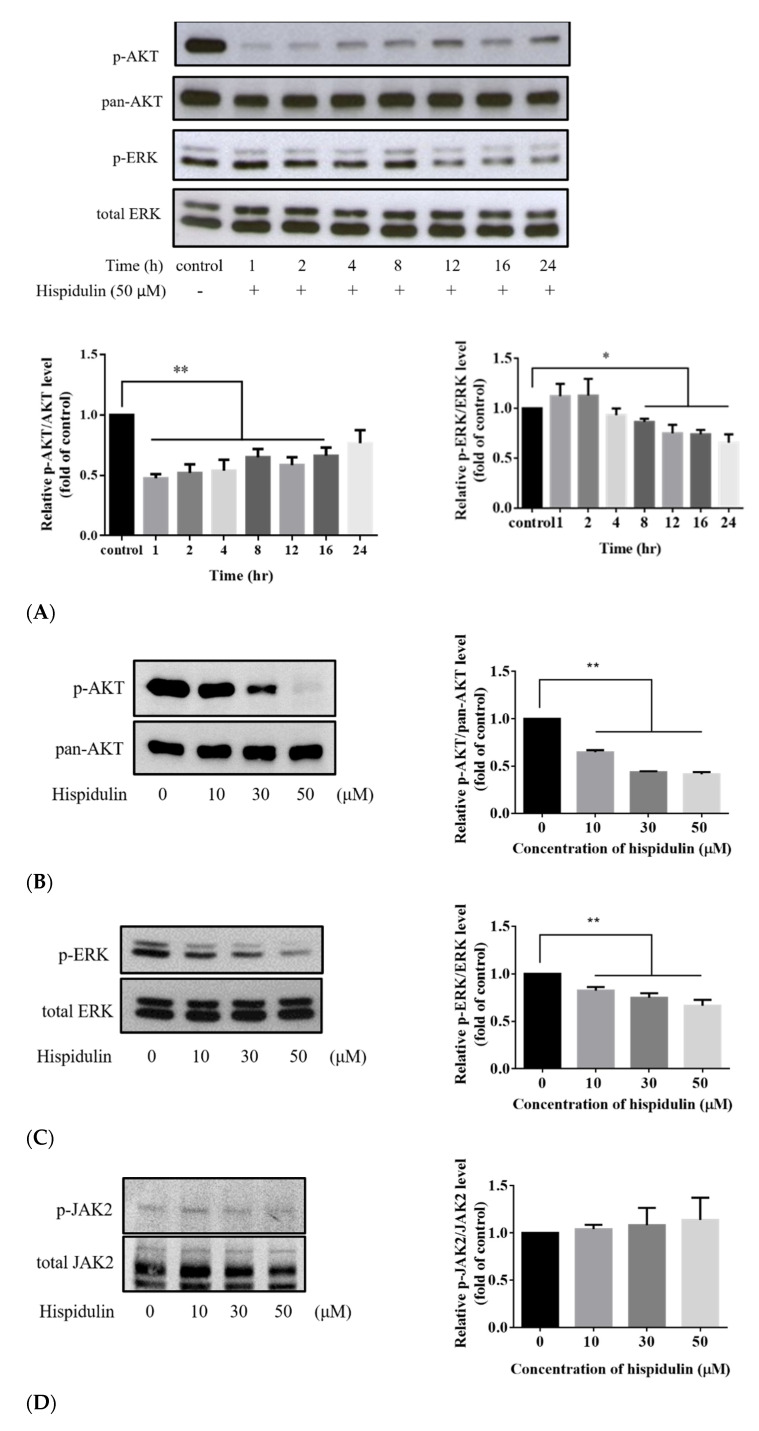

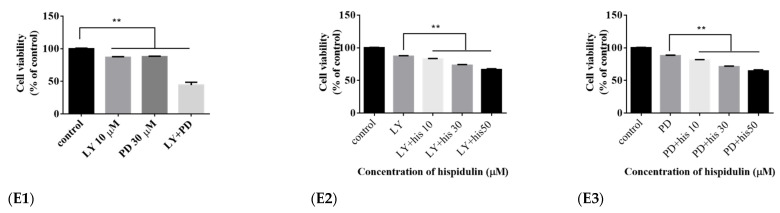
(**A**) Time course for hispidulin-inhibited AKT and ERK phosphorylation in A2058 cells. The A2058 cells were treated with 50 µM of hispidulin for the time course (upper panel). The data were normalized to the p-AKT or p-ERK level of control group (lower panel). The levels of p-AKT and p-ERK were determined by Western blot analysis. Equal loading of total proteins in each sample were verified by pan-AKT and total ERK expression. The A2058 cells were incubated with various concentrations of hispidulin (0, 10, 30, and 50 µM) for the indicated time. The levels of (**B**) p-AKT (after 1 h), (**C**) p-ERK (after 24 h), and (**D**) p-JAK2 were determined by Western blot analysis. Equal loading of total proteins in each sample were verified by pan-AKT, total ERK, and total JAK2 expression. The intensity of the bands obtained were quantified using the ImageJ software. (**E1**–**E3**) A2058 cells pretreated with LY294002 (LY; AKT inhibitor), PD98059 (PD; ERK inhibitor). or hispidulin for 24 h and cell survival as determined by the MTT assay. Results are expressed as percentage of control and mean ± SE, with ** *p* < 0.01 compared with the untreated control group (n = 5).

Mitogen-activated protein kinase (MAPK)/ERK and AKT pathways have been extensively studied and heavily targeted by drugs to treat metastatic melanoma [27]. Hispidulin is a multitarget natural anticancer substance, and the main targets involved in different cancers are also different, but the AKT pathway and the ERK pathway may be the main regulatory points for hispidulin to inhibit cancer cell growth [11,13]. When the A2058 cells were treated with a high concentration of hispidulin for 1, 2, 4, 8, 12, 16, and 24 h, the expression of p-AKT was decreased. A similar effect was also found in p-ERK. The decrease of p-AKT at 1 h is the most significant, and p-ERK has the most obvious effect at 24 h (Figure 6A). When different concentrations (0, 10, 30, and 50 µM) of hispidulin were treated to A2058 cells, the expression levels of p-AKT and p-ERK also varied in a dose-dependent manner (Figure 6B,C). In contrast to decreasing p-ERK and p-AKT, we found that hispidulin did not affect the protein level of p-JAK2 in A2058 cells (Figure 6D). AKT inhibitor LY294002 or ERK inhibitor PD98059 was used to further verify that hispidulin reduced cell viability of A2058 cells by inhibiting p-AKT and p-ERK expressions. The results showed that the treatment of LY294002 (10 µM) or PD98059 (30 µM) can significantly reduce the cell viability of A2058 cells (Figure 6E). At this time, the cell viability will be more reduced after treatment with PD98059 and LY294002 together (Figure 6E1). These results indicated that the signal pathways of AKT and ERK are independent. On the other hand, the results showed that LY294002 (10 µM) can significantly reduce the cell viability of A2058 cells (86.79 ± 1.05%), whereas the cell viability decreased to 82.83 ± 0.6%, 73.23 ± 1.05%, and 66.57 ± 1.22% after co-treatment of LY294002 (10 µM) and different hispidulin concentration (10, 30, and 50 µM) (Figure 6E2). The results showed that PD98059 (30 µM) can significantly reduce the cell viability of A2058 cells (87.75 ± 0.87%), whereas the cell viability decreased to 80.98 ± 0.88%, 70.96 ± 0.97%, and 64.58 ± 1.59% after co-treatment of PD98059 (30 µM) and different hispidulin concentration (10, 30, and 50 µM) (Figure 6E3). Therefore, these results indicate that hispidulin may reduce the phosphorylation of AKT and ERK and contribute to the reduction of cell viability of A2058 cells.

### 3.5. Hispidulin Induced Generation of ROS in A2058 Cells

**Figure 7:**
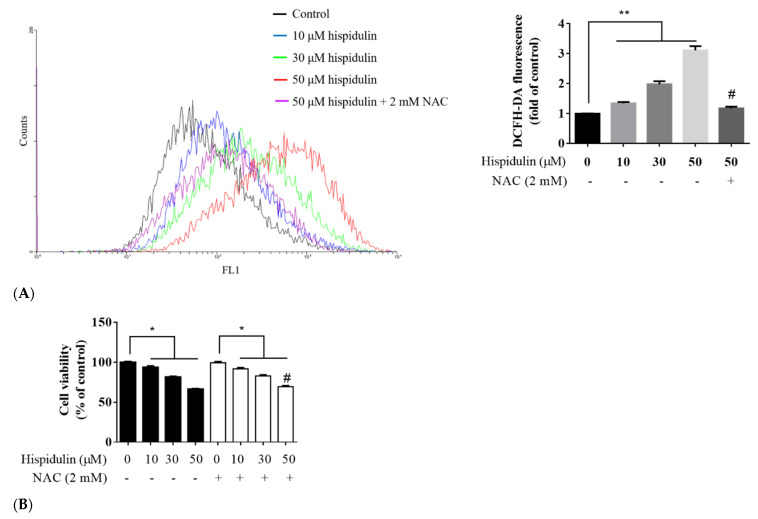
(**A**) Effect of hispidulin on ROS generation in A2058 cells. Detection of intracellular ROS level. A2058 cells were treated with different concentrations (0, 10, 30, and 50 µM) of hispidulin for 24 h or pretreated with 2 mM NAC for 1 h, and intracellular ROS levels were measured by flow cytometry (after staining with DCFH-DA reagent). Data are presented as fold of control and mean ± SE, with ** *p* < 0.01 compared with the untreated control group, and # *p* < 0.01 compared with the group treated with 50 µM hispidulin alone. (**B**) The effect of pretreatment with antioxidant on the death of A2058 cells induced by hispidulin. A2058 cells pretreated with 2 mM NAC for 1 h then incubated with indicated concentration of hispidulin for 24 h, or treated with hispidulin alone and determined cell viability by MTT assay. Data are presented as percentage of control and mean ± SE, with * *p* < 0.05 and ** *p* < 0.01 compared with the control group, and # *p* < 0.05 compared with the group treated with 50 µM hispidulin alone (n = 6).

Using DCFH-DA to detect intracellular ROS, ROS production increased in a dose-dependent manner after treatment with hispidulin (10–50 µM) for 24 h. When A2058 cells were pre-treated with 2 mM antioxidant-N-acetylcysteine (NAC) for 1 h and then treated with hispidulin, the ROS production significantly reduced, relative to the group treated with hispidulin only (Figure 7A). These results suggest that hispidulin induces the production of ROS in A2058 cells. Furthermore, we analyzed the effect of hispidulin-induced ROS on cell viability of A2058 cells. The cells were pre-treated with 2 mM NAC for 1 h and then treated with different concentrations (10, 30, and 50 µM) of hispidulin for 24 h. Under treatment of hispidulin alone, the cell viability decreased significantly in a dose-dependent manner. For the group pre-treated with NAC, the cell viability was 99.34 ± 1.35%, 91.97 ± 1.31%, 82.96 ± 1.25%, and 69.39 ± 1.16%, respectively (Figure 7B). Comparing the group treated only with the concentration of 10 and 30 µM hispidulin, the cell viability did not recover, while the group with the concentration of 50 µM hispidulin showed a slight recovery. Overall, hispidulin-induced ROS production in A2058 cells may not be directly related to cell death.

### 3.6. Hispidulin Suppressed A2058 Cells’ Migration

**Figure 8:**
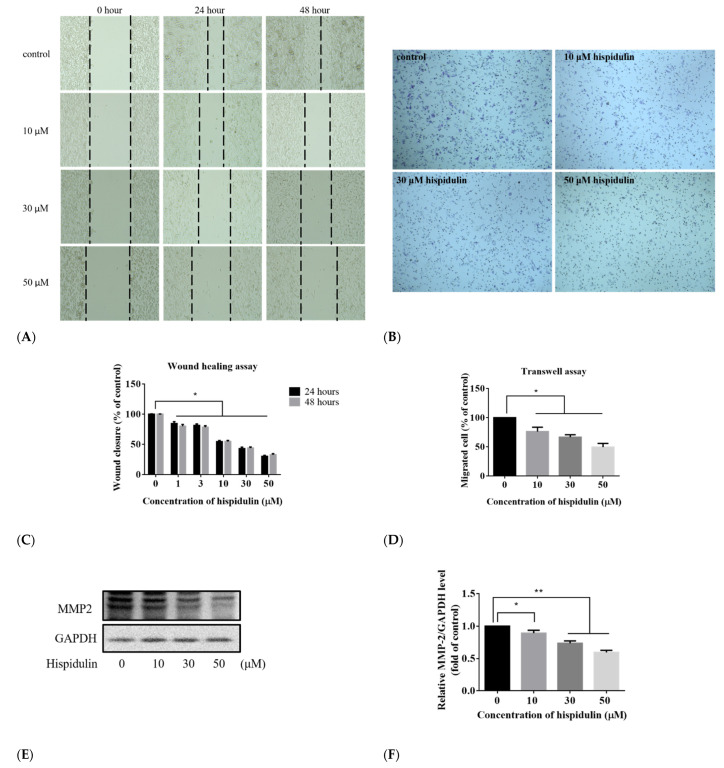
Hispidulin suppressed the motility of the A2058 cells through the wound healing assay and transwell assay (**A**,**B**). Cell mobility was determined by the wound healing assay. Monolayers of A2058 cells were treated with various concentrations of hispidulin for 24 and 48 h. Cell mobility was determined by the transwell chamber assay. A2058 cells were pretreated with different concentrations of hispidulin for 24 h then seeded in the upper chamber for 5 h. After incubation for 5 h at 37 °C, fixation was performed, and non-migrated cells were removed. A2058 cells that migrated to the underside of filter membrane were photographed and counted. (**C**) The quantitative assessment of the number of cells in the denuded zone. (**D**) A2058 cells that migrated to the underside of filter membrane were photographed and counted. (**E**,**F**) The expression levels of MMP-2 in A2058 cells treated with different concentrations (0, 10, 30, and 50 µM) of hispidulin for 24 h and examined by Western blot assay. Data were presented as fold of control and mean ± SE, with * *p* < 0.05 and ** *p* < 0.01 compared with the untreated control group (n = 5).

We evaluated the effect of hispidulin on the migration of A2058 cells and explored it through the scratch wound healing assay and transwell assay. In the wound healing assay, A2058 cells moved significantly to the exposed area in the control group. When the cells were treated with hispidulin (1–50 µM), the migration of A2058 cells was significantly inhibited. The inhibitory effect was also dose-dependent. After 24 h of treatment, the wound healing rates in the control group and treatments of hispidulin groups were 100 ± 0.58%, 84.50 ± 3.24%, 81.37 ± 2.49%, 54.52 ± 1.97%, 43.03 ± 2.42%, and 30.37 ± 1.6%, respectively. When treated with 30 µM hispidulin, the inhibitory ratio was more than 50% higher than that of the control group (Figure 8A,C). In the transwell assay, it can also be found that the migration of A2058 cells is significantly inhibited compared with the control group (Figure 8B,D). These results suggested that hispidulin has the activity to inhibit A2058 cell migration. Degradation of extracellular matrix (ECM) is highly correlated with cancer cell migration and invasion. When A2058 cells were treated with different concentrations of hispidulin (0, 10, 30, and 50 µM), the expression of MMP-2 significantly decreased in a dose-dependent manner (Figure 8E). It shows that hispidulin may inhibit A2058 cell migration by downregulating MMP-2 expression.

### 3.7. Hispidulin Restrained Tumor Growth in a Xenograft Model

**Figure 9:**
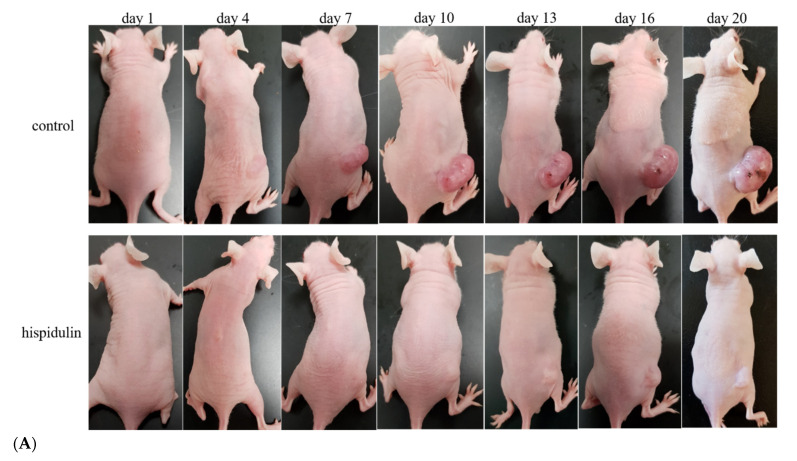

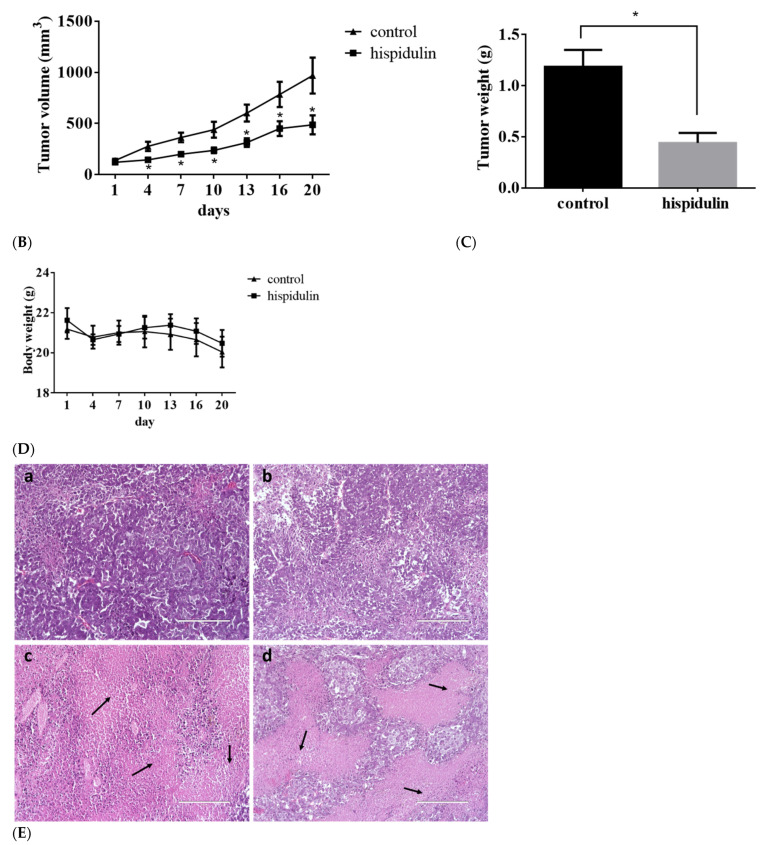
Hispidulin inhibited tumor growth in the xenograft model. A2058 cells inoculated subcutaneously into the nude mice. After a week, mice then received intraperitoneal (i.p.) injections of the vehicle (DMSO in PEG 400) or 40 mg/kg of hispidulin once per day for 19 consecutive days. On the 20th day, the mice were euthanized. (**A**) Representative mice with A2058 xenografts are shown at different time-points. Day 1 represented the first day of administration and mice were recorded every 3 days. (**B**) Time-dependent effect of hispidulin on melanoma growth in an A2058 xenograft mouse model. (**C**) Tumor weight measured at the endpoint and typical photographs. (**D**) Body weights at different time points. The data represent the mean ± SE, with * *p* < 0.05 compared with the control group (vehicle). (**E**) The H&E staining analyzed tumor tissue sections from the mice treated with (**a**,**b**) vehicle or (**c**,**d**) hispidulin (magnification, 200×) (n = 7 per group). Arrows indicate the large areas of tumor cell necrosis.

Xenotransplantation of A2058 cells into nude mice was used to evaluate the anti-melanoma effect of hispidulin in vivo. A2058 cells were implanted in mice by subcutaneous injection. After one week, mice (tumor volume reached 100–200 mm^3^) were administered an intraperitoneal injection of vehicle (DMSO in PEG 400) or hispidulin 40 mg/kg for 19 consecutive days. The tumor volume and body weight of mice were measured every 3 days. Compared with the control group, the tumor volume of mice administered hispidulin was significantly inhibited. The tumor volume of the final control group and the hispidulin group were 969.06 ± 175.72 and 486.68 ± 92.48 mm^3^, respectively. The tumor weight of mice administered with hispidulin was significantly lower than that of the control group on the 20th day after continuous drug administration. The tumor weight of the control group was 1.18 ± 0.17 g, while the weight of the hispidulin group was 0.44 ± 0.1 g (Figure 9A–C). The body weight was recorded daily to determine whether treatment affected the health of the animals [28]. The weight of mice administered hispidulin did not change significantly (Figure 9D). This indicated that hispidulin may have lower side effects on mice. The control group and hispidulin group had no abnormalities with regards to other organs of the mice, including their lungs, liver, and heart. Furthermore, the clinical symptoms (fur, eyes, excreta) of mice were also observed daily without any abnormal states (data not shown). In addition, the type of melanoma cells can be distinguished by the special enlargement of the nucleus, irregular borders, abundant cytoplasm, and vacuole formation. We found that the number of typical cancer cells in the control group was significantly higher than that in to hispidulin group (Figure 9E).

## 4. Discussion

The high mortality rate of melanoma is due to its high invasiveness, metastaticity, and multiple drug resistance. The current treatment is mainly surgical resection, but it is limited to carcinoma in situ. Other treatments such as immunotherapy, target therapy, chemotherapy, and radiation therapy are often accompanied by side effects and drug resistance [29]. Therefore, the average survival rate is still not significantly improved. Developing effective drugs with few side effects is necessary. Hispidulin has been found to have anticancer activities in various cancers, such as glioblastoma multiforme, gastric cancer, colorectal cancer, liver cancer, pancreatic cancer, gallbladder cancer, renal cell cancer, and ovarian cancer [11,12,13,14,16,18,20,24,26]. Therefore, we speculated that hispidulin must also have considerable anticancer activity in melanoma. Indeed, a series of experiments have now confirmed our point of view. Previous studies have shown that the dysregulation of the PI3K signal pathway is common in melanoma [30], which triggers the phosphorylation of AKT which, in turn, promotes melanoma cell survival, growth, and anti-apoptotic reactions. Some reports also speculate that the degree of AKT phosphorylation is negatively correlated with patient survival [31]. Hispidulin is a multitarget natural anticancer substance, and the main targets involved in different cancers are also different, but the AKT pathway and the ERK pathway may be the main regulatory points for hispidulin to inhibit cancer cell growth [11,13]. Gao et al. found that hispidulin induces apoptosis through mitochondrial dysfunction and inhibition of the PI3K/AKT signaling pathway in HepG2 cancer cells [25]. In the present experiment, we found that hispidulin can reduce AKT phosphorylation in a dose-dependent manner and AKT inhibitor LY294002 promotes cell death of melanoma. In addition, abnormal activation of MAPK/ERK in melanoma is also a common event [30]. This pathway regulates cell growth, proliferation, and survival. Han et al. indicated that hispidulin inhibits hepatocellular carcinoma growth and metastasis through AMPK and ERK signaling-mediated activation of PPARγ [31]. Similarly, the results of this study show that hispidulin can reduce ERK phosphorylation, and ERK inhibitor PD98059 leads to increased death of A2058 cells (Figure 6E). These experiments suggest that hispidulin reduces the viability of A2058 cells by reducing the phosphorylation of AKT and ERK. However, the role of downstream and upstream molecules of these signaling pathways needs to be further studied in detail in the future.

ROS is highly correlated with cell growth, apoptosis, migration, and invasion ability. ROS interacts with the Bcl-2 and p38 to activate the endogenous apoptosis pathway. Furthermore, ROS stops the cell cycle by affecting various checkpoints, which leads to the inhibition of cell growth [32]. Previous studies have indicated that hispidulin inhibits the Janus kinase 2 (JAK2)/STAT3 pathway by generating ROS in colorectal cancer cells, and downregulates the expression of the oncogene PIM1. However, treatment of NAC can slow down hispidulin-induced apoptosis and inhibit cell invasion [33]. In this study, pre-treatment with 2 mM NAC did not restore the phenomenon that hispidulin induces apoptosis of A2058 cells. It has been shown that ROS induced by hispidulin is not directly related to cell viability in A2058. Previous literature has demonstrated that STAT3 is abnormally activated in a variety of human cancers. It can promote cancer progression by inhibiting cancer cell apoptosis, promoting cell proliferation, angiogenesis, invasion, and metastasis. STAT3 can be activated by upstream tyrosine kinase Src and JAK. Our results showed that phospho-JAK2 was not affected by treatment of hispidulin in our study (Figure 6D). Therefore, whether hispidulin affects Src or other JAK family proteins and affects the STAT3 signaling pathway, which in turn affects downstream gene expression, requires further research in the future.

Metastasis is the main cause of melanoma’s high mortality rate. Metastasis goes through a series of molecular mechanisms, from cancer cells to loss of adhesion, migration, invasion of stromal tissue, degradation of extracellular matrix, penetration of blood vessels, and metastasis to distant organs [34]. Therefore, inhibiting the migration of cancer cells is one of the important topics of anti-cancer effects. Our study uses the wound healing assay and transwell assay to evaluate the effect of hispidulin on the migration ability of A2058 cells. The results show that hispidulin can inhibit the migration of A2058 cells. However, hispidulin at a concentration of more than 10 µM is cytotoxic to A2058. In the wound healing assay, the cell death caused by hispidulin cannot be avoided when observing cell migration. However, after deducting the cell death induced by hispidulin, it can still be found that hispidulin has the effect of inhibiting cell migration, and its inhibition rate is about 20–35%. In addition, its ability to inhibit cell migration was evaluated with 1 and 3 µM low-toxic hispidulin, and the inhibition rate was up to 20% (Figure 8C), showing that low concentrations of hispidulin also exhibited the effect of inhibiting A2058 cell migration. In the transwell assay, it was also found that hispidulin can inhibit the migration of A2058 cells, and its inhibition rate is dose-dependent. Matrix metalloproteinases are zinc-dependent endopeptidases, which participate in physiological ECM degradation and are an important step in cancer cell metastasis. MMP-2 and MMP-9 play a key role in cancer migration and invasion. The expression of matrix metalloproteinases can effectively reflect the invasion ability of cancer cells [35]. Therefore, this study explored the effect of hispidulin on the expression of MMP-2 and MMP-9 in A2058 cells. In A2058 cells treated with hispidulin, the expression levels of MMP-2 and MMP-9 both decreased. Among them, the decrease of MMP-2 has a dose-dependent effect, while the expression of MMP-9 has a recovery phenomenon at a high concentration of 50 µM (data not shown). It is speculated that hispidulin can inhibit the migration of A2058 cells by downregulating MMP-2 and MMP-9. The literature also points out that PI3K/AKT and MAPK/ERK can regulate the performance of downstream Snail, which is the zinc-finger transcription factor and the main regulator of epithelial–mesenchymal transition (EMT). When Snail decreases cell adhesion factors (E-cadherin), the cells lose their adhesion and their polarity [36], and then cells will metastasize and invade to another organ. Therefore, the expression of AKT or ERK was inhibited and cell migration ability could be decreased [37]. In this study, the inhibition of AKT and ERK phosphorylation may also be involved in the event that hispidulin inhibits A2058 cell migration. In addition, it is known that ultraviolet radiation is one of the main environmental carcinogens of melanoma, and previous studies have shown that hispidulin has anti-UVA-induced photoaging effects [38]. It shows that hispidulin also has a photoprotective effect, and the photoprotective mechanism of hispidulin still needs to be discussed in detail.

Among the flavonoid members, apigenin (5,7,4′-Trihydroxyflavone) is widely found in plants, such as *Lycopodium clavatum* L., *Petroselinum crispum* L., and *Apium graveolens* L. Studies have confirmed that apigenin can resist DNA damage and inflammatory diseases caused by UVB. In cancer, apigenin has anti-proliferation, cell cycle arrest, and activation of ataxia telangiectasia-mutated (ATM) and H2AX-induced cancer cell apoptosis [39]. Hispidulin and apigenin are both flavonoids, and the structure is similar. The two differ only by one methoxy group at the C6 position (–OCH3). Zhao et al. found that apigenin can inhibit the proliferation and invasion of human melanoma cancer cell lines. Apigenin induces cell apoptosis through the activation of caspase-3 and PARP, promotes cell cycle arrest in the G2/M phase, and can inhibit AKT/mTOR and ERK signaling pathways [39]. Woo et al. indicated that apigenin induces apoptosis by regulating AKT and MAPK pathways in human melanoma cell A375SM [40]. The above mechanisms of action are similar to hispidulin. These results indicate that natural flavonoids may be developed as anti-melanoma therapeutic agents in the future.

## 5. Conclusions

From in vitro experiments of hispidulin against melanoma, it was found that hispidulin effectively activates caspase 8 and caspase 9, as well as increases the expression of cleaved caspase 3 and cleaved PARP. It was shown that hispidulin induces A2058 cell apoptosis through exogenous and endogenous pathways. It promotes the accumulation of cells in the sub-G1 phase, and the cell cycle arrest in the S and G2/M phases inhibits cell growth. However, hispidulin mainly induces apoptosis rather than autophagy in A2058 cells. Hispidulin is selectively cytotoxic to A2058 cells and has low toxicity to normal keratinocytes. Hispidulin can reduce the phosphorylation of AKT and ERK, thereby reducing the cell survival rate. Hispidulin may reduce the expression of MMP-2 and reduce migration of A2058 cells. In addition, hispidulin can promote the production of ROS in A2058 cells. In the A2058 xenograft model, hispidulin can inhibit tumor growth, but has low side effects in mice. In summary, hispidulin has potential as an anti-melanoma agent.

## Data Availability

Not applicable.
